# Lactate Dehydrogenase B Expression in Cerebellum of Adult *Ube3a* ^m-/p+^ Mice

**DOI:** 10.17912/micropub.biology.001999

**Published:** 2026-05-04

**Authors:** Miriam Arlene Ramos Rivera, Ceidy Torres Ortiz

**Affiliations:** 1 Department of Biomedical Sciences , Pontifical Catholic University of Puerto Rico, Ponce, Puerto Rico; 2 Department of Natural Sciences, Pontifical Catholic University of Puerto Rico, Ponce, Puerto Rico

## Abstract

Angelman syndrome (AS) is a neurodevelopmental disorder caused by the loss of the maternal allele of the *UBE3A* gene, which encodes a protein essential for ubiquitin-mediated protein degradation. AS mouse models exhibit elevated lactate and acetate levels and increased *Ldha* gene expression in fibroblasts. We hypothesize that maternal *Ube3a* loss alters the protein levels of Ldha, Ldhb, Mct2, and Mct1, contributing to elevated brain lactate levels. Western blot analysis of the cerebellum from adult *Ube3a* ^m-/p+^ AS and wild-type mice reveals significant sex- and genotype-specific differences in Ldhb expression. Loss of Ube3a alters the expression of Ldhb in the cerebellum.

**Figure 1. Ldha, Ldhb, Mct2, and Mct1 Protein Expression in the Cerebellum and Hippocampus:** Note: Western Blots a, b, c, and d show antibody bands for β-Actin (45 kDa), Ldha (37 kDa), Ldhb (36 kDa), Mct2 (52 kDa), and Mct1 (51 kDa) in the cerebellum, while m, n, and o show these in the hippocampus. In the gel, the samples are intercalated, starting with WT, then AS; males (1-10 wells position), and females (11-20 wells position). All mice are adults between 10 to 12 week old. Graphs e, f, g, and h show protein expression of Ldha, Ldhb, Mct2, and Mct1 in the cerebellum, while p, q, r, and s show these in the hippocampus. Graphs p and t correspond to Mct2, and graphs q and u correspond to Ldha in the hippocampus. (f) Student's t-test ( *N* =20) found a significant difference in Ldhb fold change between AS and WT ( *p* =0.0113). Graphs i, j, k, and l compare gender and genotype using Two-way ANOVA in the cerebellum, while t, u, v, and w show these in the hippocampus (WT = Green, AS = Purple). There are no significant differences in (i, k, l, t, u, v, and w). (j) Two-way ANOVA reports a significant Ldhb difference in cerebellum males ( *p* =0.0128, *n* =10 per group).

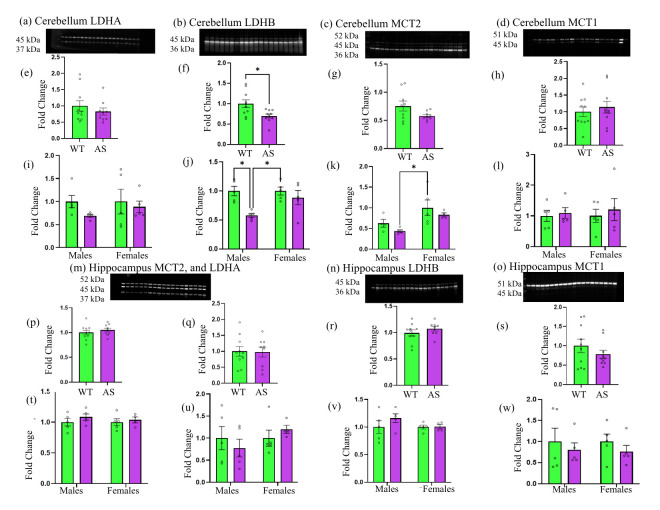



## Description

Ubiquitin Protein Ligase E3A (UBE3A) is vital for nervous system development, neuron maturation, synaptic plasticity, and brain growth (Leader et al., 2022; Bird, 2014). Only in neurons does this gene undergo maternal imprinting defects or deletions; consequently, loss of the maternal allele results in Angelman Syndrome (AS), a well-characterized neurodevelopmental disorder (Dagli et al., 1998; Sun et al., 2019). Recent studies report alterations in lactate metabolism in AS, including increased Lactate dehydrogenase A ( *Ldha* ) gene expression in embryonic fibroblasts of the AS model (Simchi et al., 2020) and elevated lactate levels in lyophilized AS brain samples (Gupta et al., 2024). According to the astrocyte–neuron lactate shuttle hypothesis, astrocytes produce lactate from glucose via LDHA, which is transported into neurons through Monocarboxylate Transporter (MCT2) and converted back to pyruvate by LDHB to support neuronal energy metabolism (Suzuki et al., 2011; Liu et al., 2017; Medel et al., 2022; Kim et al., 2025). Also, it serves as a signaling molecule in various mechanisms, including the regulation of energy metabolism, immunological responses, memory formation, and muscle contraction (Li et al., 2022).

In Alzheimer’s disease models, lower hippocampal lactate levels correlate with memory impairment (Lu et al., 2018), and altered expression of lactate transporters (MCT1, MCT2, MCT4) indicates impaired lactate signaling in neurological disorders (Wang et al., 2019). In the cerebellum, which regulates motor coordination, posture, and balance, disruptions in the lactate shuttle impair motor performance, as shown by reduced function upon Mct2 inhibition in mice (Hoshino et al., 2016; Pierre and Pellerin, 2005; Li et al., 2022). Studying lactate pathways in AS helps to understand how elevated lactate affects the adult brain. This study examines Ldha, Ldhb, Mct2, and Mct1 levels in the hippocampus and cerebellum of *Ube3a* ^m-/p+^ (AS) compared with wild-type (WT) mice. We hypothesize that maternal Ube3a loss affects the expression of Ldha, Ldhb, Mct2, and Mct1, possibly contributing to elevated lactate levels in AS. The protein expression fold changes of Ldha, Ldhb, Mct2, and Mct1 were assessed by Western blot in hippocampal and cerebellar tissues from adult male and female AS and WT mice. The data were analyzed using a Student’s t-test to compare genotypes and a two-way ANOVA to assess sex differences across genotypes.

In the hippocampus, no significant differences were observed in Ldha, Ldhb, Mct2, or Mct1 expression between AS and WT mice ( **Fig.1 p-s** ). Also, any sex-specific differences in protein expression between AS and WT mice ( **Fig.1 t-w** ). Conversely, Ldhb expression in the cerebellum was significantly lower in AS mice ( *p* = 0.0113, **Fig.1 f** ) while Ldha, Mct1, and Mct2 showed no significant differences ( **Fig. e, g, h** ). The reduced Ldhb expression in the cerebellum of AS mice is attributable to the male group (p=0.0128, **Fig. j** ). These results suggest that reduced Ldhb may impair lactate-to-pyruvate conversion, decreasing pyruvate availability, elevating lactate levels, and disrupting the TCA cycle, ultimately leading to bioenergetic deficits and motor impairments in the cerebellum, particularly in adult males. These results confirm previous reports that AS mice exhibit motor deficiencies in motor tasks, such as decreased grip strength and performance on the raised beam task (Heck et al., 2008), and higher latency to fall on the rotarod test (Sun et al., 2015). The loss of maternal UBE3A in the adult *Ube3a* ^m-/p+^ AS mouse model affects Ldhb protein expression in the cerebellum but not in the hippocampus. This suggests that the absence of maternal UBE3A may specifically influence the expression of specific metabolic proteins in the cerebellum. Observations suggest lactate may increase during early development in AS mice (Gupta et al., 2024), possibly affecting LDHs and MCTs in adulthood. These studies contribute to our understanding of lactate metabolism in AS and highlighting the need for continued research given the limited number of reports in this field.

## Methods

**Western Blot Analysis:**

The hippocampus and cerebellum frozen tissues were homogenized in 300 μL RIPA buffer (50 mM Tris-HCl, 150 mM NaCl, 1% NP-40, 0.5% sodium deoxycholate, 0.1% SDS) supplemented with protease and phosphatase inhibitors (Pierce TM Protease and Phosphatase Inhibitor Mini Tablets, Thermo Scientific). The protein concentration was determined using the Qubit Protein BR Assay Kit (Invitrogen, now Thermo Fisher Scientific) as per the manufacturer’s instructions. The protein was denatured with 5X Laemmli sample buffer (10% sodium dodecyl sulfate, 25% 2-mercaptoethanol, 30% glycerol, 0.05% bromophenol blue, 292 mM Tris HCl pH 6.8) at 95°C for 10 minutes before loading onto a 4-15% gradient polyacrylamide gel (BIO-RAD Criterion™ TGX™ Precast Gels) and electrophoresing at 80V in 1X running buffer (25 mM Tris, 192 mM glycine, 0,1% SDS, pH 8.3) for 1.5 hours. 10 µg was loaded to visualize the expression of Ldha, Ldhb, Mct1 and Mct2. The proteins were transferred to a 0.2 µm PVDF membrane at 25V for 7 minutes in 1X transfer buffer (Trans-Blot Turbo 5x Transfer Buffer, BIO-RAD) using the Trans-Blot Turbo system (BIO-RAD). The membranes were blocked with 5% bovine serum albumin (BSA) in 50 mL of TBST for 1 hour at room temperature. Primary antibodies ( **Table 1** ) were diluted in TBST and incubated overnight at 4 °C on a shaker at 60 rpm. After incubation, the membranes were washed three times with 10 mL TBST for 10 minutes each. Secondary antibodies ( **Table 1** ) were diluted in TBST and incubated for 1 hour at room temperature on the shaker. Protein detection was performed using the SuperSignal™ West Pico Plus Chemiluminescent Substrate (Thermo Fisher Scientific). Equal volumes of enhancer solution and stable peroxide solution were applied to the membrane and incubated on a shaker at 60 rpm for 5 minutes, after which the membrane was imaged.

**Statistical analysis:**

The membrane images were analyzed using ImageJ-win 64 to measure the intensity of the antibody bands. Then, the data was normalized by dividing the band of interest by the band of the housekeeping gene, β-Actin. Next, we averaged the normalized expression of the WT group (WT mean). Finally, we divided the normalized expression values for the WT and AS groups by the WT mean to obtain fold changes. The fold-change data were analyzed using a Student’s t-test to compare WT and AS across the different tissues. A two-way analysis of variance (ANOVA) was used to compare genotype and sex-specific differences.

## Reagents

**Table:** 

**Table 1: Strain Description and Antibodies Information**					
STRAIN	GENOTYPE	AVAILABLE FROM			
*Ube3a* ^m-/p+^	C57BL/6J	The Jackson Laboratory			
*Ube3a* ^m+/p+^	C57BL/6J	The Jackson Laboratory			
ANTIBODY	DILUTION	ANIMAL AND CLONALITY	DESCRIPTION		
anti-Lactate dehydrogenase A	5:10,000 in TBS-0.1% Tween 20	Rabbit polyclonal	Cell Signaling Technology		
anti-Lactate dehydrogenase B	1:10,000 in TBS-0.1% Tween 20	Rabbit polyclonal	Bethyl Laboratories		
anti-Monocarboxylate Transporter 2	1:10,000 in TBS-0.1% Tween 20	Rabbit polyclonal	EMD Millipore Corp		
anti-Monocarboxylate Transporter 1	1:10,000 in TBS-0.1% Tween 20	Rabbit polyclonal	Thermo Fisher Scientific		
anti-Beta Actin	1:10,000 in TBS-0.1% Tween 20	Rabbit polyclonal	Cell Signaling Technology		
Goat anti-rabbit IgG(H+L), horseradish peroxidase conjugate	1:10,000 in TBS-0.1% Tween 20	Rabbit polyclonal	Invitrogen by Thermo Fisher Scientific		
